# Predictors of Preterm Neonatal Mortality in India and Pakistan: A Secondary Analysis of Data from PURPOSe Study

**DOI:** 10.1177/2333794X241236617

**Published:** 2024-03-13

**Authors:** Shiyam Sunder Tikmani, Sarah Saleem, Afreen Sadia, Carla M. Bann, Muhammad Hayat Bozdar, Jamal Raza, Sangappa M. Dhaded, Shivaprasad S. Goudar, Guruparasad Gowdar, Haleema Yasmin, Elizabeth M. McClure, Robert L. Goldenberg

**Affiliations:** 1 The Aga Khan University, Karachi, Pakistan; 2 Research Triangle Institute (RTI) International, Durham, NC, USA; 3 National Institute of Child Health, Napier Quarter, Karachi, Pakistan; 4 JN Medical College, Belagavi, India; 5 Bapuji Educational Association’s JJM Medical College, Davangere, India; 6 Jinnah Postgraduate Medical Center, Karachi, Pakistan; 7 Columbia University, New York, NY, USA

**Keywords:** preterm neonates, preterm neonatal mortality, India, Pakistan, predictors

## Abstract

*Objective.* To create a prediction model for preterm neonatal mortality. *Methods.* A secondary analysis was conducted using data from a prospective cohort study, the Project to Understand and Research Preterm Pregnancy Outcome South Asia. The Cox proportional hazard model was used and adjusted hazard ratios (AHR) with 95% confidence intervals (95% CI) were reported. *Results.* Overall, 3446 preterm neonates were included. The mean age of preterm neonates was 0.65 (1.25) hours and 52% were female. The preterm neonatal mortality rate was 23.3%. The maternal factors predicting preterm neonatal death was any antepartum hemorrhage, AHR 1.99 (1.60-2.47), while neonatal predictors were preterm who received positive pressure ventilation AHR 1.30 (1.08-1.57), temperature <35.5°C AHR 1.18 (1.00-1.39), and congenital malformations AHR 3.31 (2.64-4.16). *Conclusion.* This study identified key maternal and neonatal predictors of preterm neonatal mortality, emphasizing the need for targeted interventions and collaborative public health efforts to address disparities and regional variations.

## Introduction

The neonatal mortality rate remains high in many low- and middle-income countries (LMIC).^1^ Preterm birth, birth asphyxia, and neonatal infections are the commonly reported causes of neonatal mortality.^2,3^ Preterm neonates are at a 2.7 times higher risk for neonatal mortality compared with full-term births in LMIC.^4-6^ Globally, in 2020, an estimated 13.4 million neonates were born before 37 weeks of gestation making up to 10% of all births. More than 80% of preterm occur in Asia and sub-Saharan Africa.^7^ Among Asian countries, Pakistan and India bear the highest burden of preterm neonatal mortality. In 2019, the preterm-related neonatal mortality rate per 1000 live births in Pakistan was 18.69 (95% confidence interval (CI): 13.52-24.51), and in India, 14.71 (95% CI: 11.78-18.19).^4^

Although progress has been made in decreasing child mortality, the number of deaths attributable to preterm birth remains disproportionately high in the south Asian region.^8^ These deaths are caused by various maternal, neonatal, and placental factors.^9^ Timely interventions like antenatal corticosteroids, kangaroo mother care, assisted ventilation, and antibiotics can improve neonatal survival. Understanding predictors of neonatal mortality in regional contexts is essential for developing effective interventions to reduce neonatal deaths.

Prediction models for neonatal mortality are used to estimate risk by combining various risk factors for clinical decision-making and improving the management of high-risk neonates. Studies have identified several predictors for time to neonatal death such as male neonate, respiratory distress syndrome, neonatal sepsis, gestational age (GA) <28.0 weeks, low Apgar score, home delivery, jaundice, maternal hypertension, and diabetes.^10-12^ However these prediction models for neonatal mortality are mostly limited to neonates admitted to a neonatal intensive care unit (NICU) in high-income countries where neonatal mortality is low.^12,13^ While, predictive models for neonatal mortality in LMICs are scarce, where preterm neonatal mortality is high. Furthermore, prediction models of neonatal mortality from LMICs are often limited to neonates who died in the NICU.^14^ Therefore, these prediction models have limited generalizability to other LMICs.^15^ From the available models few only considered vital sign data and lack integration of maternal and other clinical factors, limiting the accuracy of these models.^16^ A study conducted in south Asia on predicting neonatal mortality identified recall bias and missing data on certain predictors as significant limitations.^17^Addressing biases demands enhancing data collection, improving clinical data quality and availability, and standardizing measurement methods.^17^ We aim to develop a prediction model for preterm neonatal mortality using data from a prospective cohort study from large public sector referral hospitals in south Asia (Pakistan and India).

## Methods

### Study Design and Population

This secondary analysis was conducted using a dataset from a large prospective cohort study, the Project to Understand & Research Preterm Pregnancy Outcome and Stillbirth South Asia (PURPOSE study). The study was conducted in 5 public sector referral hospitals (3 from India and 2 from Pakistan) from July 1, 2018, and March 26, 2020. The detailed methodology of the primary study was published elsewhere.^18^ At the study hospitals, research staff screened all pregnant women older than 14 years in India and older than 18 years in Pakistan at the time of labor. Pregnant women with an expected or known preterm live birth were included whereas pregnant women with stillbirths, induced abortion, unknown GA, and those who declined informed consent were excluded from this analysis.

### Data Collection

After obtaining written informed consent at the time of delivery, study staff collected information including the woman’s medical and obstetric history, physical examination, and the clinical status and procedures performed during the hospital stay and verified with medical records. GA was calculated through an Android-based GA calculator with a predefined algorithm using the hierarchy of methods established by the American College of Obstetrics and Gynecology,^19^ using the reliable LMP if available, and ultrasound examination. If neither was available, a Ballard examination was done on the neonates by trained midwives.^19^ All data were then entered into the GA calculator. Based on this information, the GA calculator provided the best GA estimate.

Sick preterm infants were referred to the study hospitals. All preterm infants enrolled in the study were followed at 28 days of life to assess outcomes regardless of their admission to the NICU. Among those infants admitted to participating study hospitals, their clinical care, clinical investigations, and any conditions diagnosed during the hospital admission were documented. Neonates who were admitted at NICU and discharged, were followed on 28 days to document status of the neonates at home. In case parents are not available or migrated out, the outcome data were collected by telephone. If an infant died before 28 days of life at home or in health facilities other than study hospitals, a verbal autopsy was carried out by trained study staff at home 2 weeks after the death of the baby.

To ensure the quality of the data these were collected by the trained midwives working in the hospitals which was verified by a monitor on a day-to-day basis using medical records. Study coordinators monitor data collection once every week and arrange refresher training quarterly. Furthermore, data were entered and transmitted to the Research Triangle Institute (RTI) USA for quality assessment. RTI generated monthly progress and the edit reports.

### Variables

The outcome variable is the death of a preterm neonate at or before 28 days after birth, labeled as 1-Yes, 0-No.

The maternal predictors were: (1) maternal age, categorized into (<20, 20-25, 26-30, >30 years), (2) maternal education (No formal schooling, <5, 5-12, >12 years), (3) number of antenatal care visits (0, 1-3, 4+), (4) gravida (0, 1-3, 4+), (5) gestational age, categorized into (<28.0, 28.0-31.6, 32.0-36.6 weeks), (6) hemoglobin levels, categorized into (<7, 7-8.9, 9-10.9, 11-9) 12.9, ≥13 g/dl), (7) any hypertensive disorder (Yes, No), (8) any antepartum hemorrhage (Yes, No), (9) mode of delivery (Vaginal delivery, Cesarean section).

The neonatal factors investigated included: (1) Postnatal age in hours at the exam, mean (SD), (2) Sex, (Male, female), (3) birth weight (g) (<1000, 1000-1499, 1500-2499, ≥2500), (4) multiple birth (Yes, No), (5) Apgar score (1 minute) (0-3, 4-6, 7-10), (6) Apgar score (5 minutes) (0-3, 4-6, 7-10), (7) resuscitated with positive pressure ventilation (PPV) (Yes, No), (8) congenital malformations such as neural tube defects, omphalocele (Yes, No), (8) Temperature (°C) (<35.5, ≥35.5), (9) respiratory rate (breaths per minute) (<60, ≥60), (10) level of consciousness (Alert, Sleepy/Comatose), (11) admitted to the NICU (Yes, No),

Preterm was considered a live birth before 37 weeks of gestation. According to the World Health Organization: (1) extremely preterm (<28 weeks), (2) very preterm (28.0-31.6 weeks), and (3) moderate to late preterm (32.0-36.6 weeks).^20^

### Statistical Analysis

We analyzed data using SAS (version 9.4). Frequency and percentages were reported for maternal and neonatal factors and Chi-square tests were conducted to compare the distribution of maternal and neonatal factors among infants who died versus those who survived. Kaplan-Meier curves were used to graphically display the length of survival for neonates in each of the 2 sites (India and Pakistan). Cox proportional hazard models were used to compute unadjusted and adjusted hazard ratios (AHR) and 95% confidence intervals (CI) for mortality by maternal and neonatal factors. Furthermore, Firstly, Maternal and neonatal factors were analyzed separately, followed by a combined model building. A *P*-value of <.05 for multivariable model, while <.25 at univariate level was considered significant. For factors with multiple categories (eg, education), the factor is retained if the overall Wald chi-square test for the variable is significant. Regression models were run separately by the site (India, Pakistan) and for both sites combined. We calculated post-hoc power for each predictor which ranged from 90% to 100% (Supplemental Table 1).

### Ethical Consideration

The study was approved by the ethical review committees of Aga Khan University (5212-CHS-ERC-18), the National Institute of Child Health (IERB: 11/2018) and the National Bioethics Committee, Pakistan (Ref No: 4-87/NBC-312/18/RDC/3816), KLE Academy of Higher Education and Research, Belagavi, India (KAHER/EC/2017-18/D-2867), and J. J. M. Medical College, Davangere, India (JJMMC/IEC/02-2018). All women provided written informed consent before participating in the study. The PURPOSe Study was registered on ClinicalTrials.gov (NCT03438110). Written informed consent was obtained from each research participant, wherein we secured their permission for the publication of data from the study participants.

## Results

**Figure 1.**
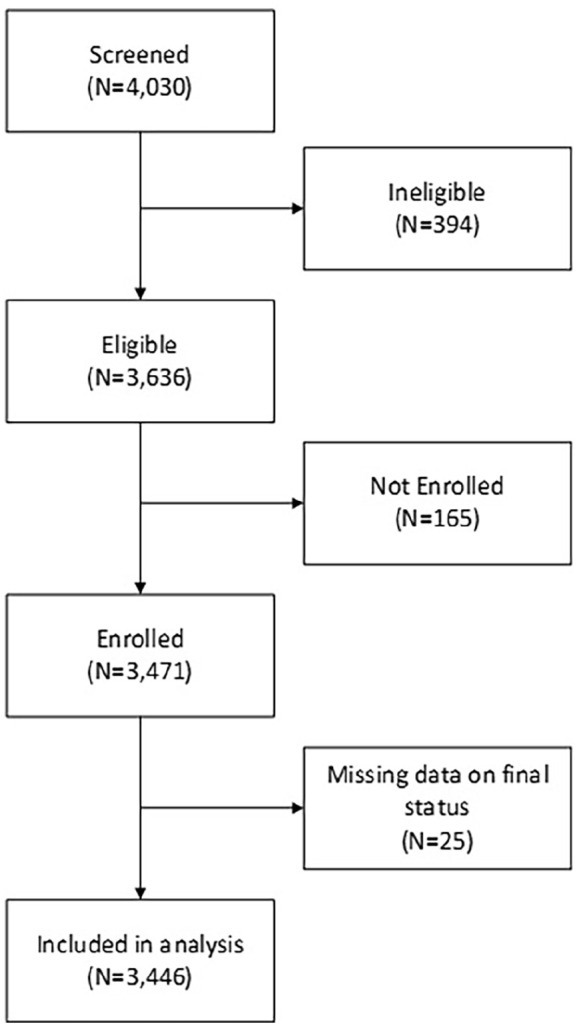
Study flow diagram.

**Figure 2.**
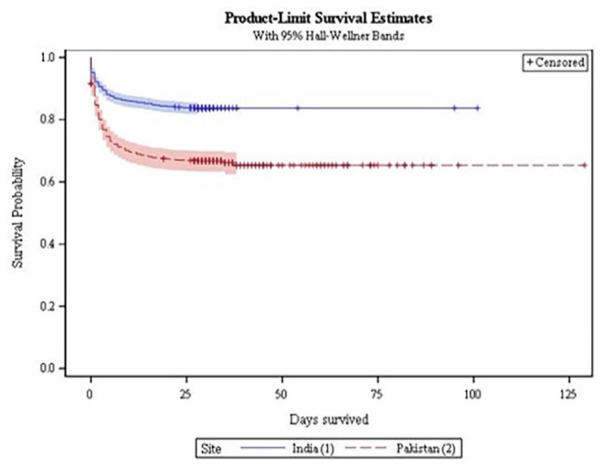
Kaplan-Meier survival curves by site.

A total of 3446 cases of preterm neonates were included in this analysis (Figure 1). Of 3446 preterm neonates, 2025 were from India and 1421 from Pakistan. Overall, 804/3446 (23.3%) preterm neonates died on or before 28 days of life. The preterm neonatal deaths were 329/2025 (16.2%) in India, while 475/1421 (33.4%) in Pakistan. The Kaplan-Meier curves clearly chart the progression of days since birth against the probability of survival (Figure 2).

Descriptives of maternal and neonatal factors by preterm neonatal outcome (died vs survived) are shown in Table 1. The proportion of preterm neonatal mortality is significantly associated with maternal age, maternal education, number of antenatal visits, gravida, GA, hemoglobin levels, any antepartum hemorrhage, and vaginal delivery. The neonatal factors include the sex of the baby, birth weight, APGAR score, multiple births, resuscitation with PPV, congenital malformation, temperature <35.5°C, level of consciousness, and NICU admission.

**Table 1.** Characteristics of Enrolled Preterm Babies, by Mortality.

Characteristics	All			India			Pakistan		
	Died (N = 804)	Survived (N = 2642)	*P*-value	Died (N = 329)	Survived (N = 1696)	*P*-value	Died (N = 475)	Survived (N = 946)	*P*-value
All infants									
Maternal factors									
Maternal age (years), n (%)									
<20	62 (8)	186 (7)	<.001	28 (9)	143 (8)	.505	34 (7)	43 (5)	.053
20-25	353 (44)	1398 (53)		179 (54)	995 (59)		174 (37)	403 (43)	
26-30	265 (33)	761 (29)		91 (28)	421 (25)		174 (37)	340 (36)	
>30	122 (15)	297 (11)		31 (9)	137 (8)		91 (19)	160 (17)	
Maternal education, n (%)									
No formal schooling	265 (33)	531 (20)	<.001	35 (11)	163 (10)	.354	230 (48)	368 (39)	.005
<5 years	31 (4)	85 (3)		16 (5)	52 (3)		15 (3)	33 (3)	
5-12 years	460 (57)	1803 (69)		246 (75)	1311 (78)		214 (45)	492 (52)	
>12 years	45 (6)	210 (8)		29 (9)	158 (9)		16 (3)	52 (6)	
Number of antenatal care visits, n (%)									
0	37 (5)	46 (2)	<.001	2 (1)	7 (0)	.851	35 (7)	39 (4)	.004
1-3	224 (28)	467 (18)		21 (6)	101 (6)		203 (43)	366 (39)	
4+	539 (67)	2119 (81)		306 (93)	1584 (94)		233 (49)	535 (57)	
Gravida, n (%)									
0	286 (36)	1055 (40)	<.001	140 (43)	766 (45)	.285	146 (31)	289 (31)	.920
1-3	403 (50)	1329 (50)		175 (53)	882 (52)		228 (48)	447 (47)	
4+	115 (14)	257 (10)		14 (4)	47 (3)		101 (21)	210 (22)	
Gestational age (weeks), n(%)									
<28.0	201 (25)	9 (0)	<.001	78 (24)	2 (0)	<.001	123 (26)	7 (1)	<.001
28.0-31.6	297 (37)	236 (9)		113 (34)	149 (9)		184 (39)	87 (9)	
32.0-36.6	306 (38)	2397 (91)		138 (42)	1545 (91)		168 (35)	852 (90)	
Hemoglobin levels (g/dl), n (%)									
<7	29 (4)	68 (3)	<.001	9 (3)	49 (3)	.492	20 (5)	19 (2)	.002
7-8.9	107 (14)	250 (10)		32 (10)	148 (9)		75 (18)	102 (12)	
9-10.9	294 (39)	923 (36)		117 (36)	530 (31)		177 (42)	393 (44)	
11-12.9	259 (34)	1042 (40)		125 (38)	724 (43)		134 (31)	318 (36)	
≥13	65 (9)	293 (11)		45 (14)	238 (14)		20 (5)	55 (6)	
Any hypertensive disorder, n (%)	247 (31)	779 (30)	.514	122 (37)	599 (35)	.551	125 (26)	180 (19)	.002
Any antepartum hemorrhage, n (%)	93 (12)	144 (5)	<.001	27 (8)	101 (6)	.125	66 (14)	43 (5)	<.001
Mode of delivery, n (%)									
Vaginal delivery	652 (81)	1698 (64)	<.001	245 (74)	997 (59)	<.001	407 (86)	701 (74)	<.001
Cesarean section	151 (19)	940 (36)		84 (26)	696 (41)		67 (14)	244 (26)	
Neonatal factors									
Postnatal age at the exam (hours), mean (SD^a^)	0.65 (0.72)	0.64 (1.48)	.981	0.17 (0.90)	0.38 (1.53)	.014	0.97 (0.21)	1.11 (1.25)	.019
Sex, n (%)									
Female	353 (44)	1300 (49)	.011	139 (42)	822 (48)	.039	214 (45)	478 (51)	.065
Male	447 (56)	1340 (51)		190 (58)	874 (52)		257 (55)	466 (49)	
Birth weight (g), n (%)									
<1000	188 (23)	15 (1)	<.001	95 (29)	13 (1)	<.001	93 (20)	2 (0)	<.001
1000-1499	288 (36)	243 (9)		135 (41)	216 (13)		153 (32)	27 (3)	
1500-2499	289 (36)	1689 (64)		88 (27)	1190 (70)		201 (42)	499 (53)	
≥2500	38 (5)	695 (26)		11 (3)	277 (16)		27 (6)	418 (44)	
Multiple birth	160 (20)	375 (14)	<.001	59 (18)	228 (13)	.033	101 (21)	147 (16)	.007
Apgar score (1 minute), n (%)									
0-3	295 (38)	84 (3)	<.001	157 (49)	67 (4)	<.001	138 (31)	17 (2)	<.001
4-6	364 (47)	1464 (57)		147 (46)	1212 (74)		217 (49)	252 (27)	
7-10	109 (14)	1021 (40)		19 (6)	357 (22)		90 (20)	664 (71)	
Apgar score (5 minutes), n (%)									
0-3	192 (25)	21 (1)	<.001	83 (26)	14 (1)	<.001	109 (24)	7 (1)	<.001
4-6	281 (36)	302 (12)		124 (38)	229 (14)		157 (35)	73 (8)	
7-10	304 (39)	2249 (87)		116 (36)	1395 (85)		188 (41)	854 (91)	
Resuscitated with PPV^b^, n (%)	283 (36)	149 (6)	<.001	168 (51)	113 (7)	<.001	115 (25)	36 (4)	<.001
Congenital malformations, n (%)	604 (75)	481 (18)	<.001	275 (84)	371 (22)	<.001	329 (69)	110 (12)	<.001
Temperature (°C), n (%)									
<35.5	257 (32)	703 (27)	.002	142 (43)	565 (34)	<.001	115 (25)	138 (15)	<.001
≥35.5	534 (68)	1907 (73)		187 (57)	1121 (66)		347 (75)	786 (85)	
Respiratory rate (breaths per minute), n (%)									
<60	126 (16)	293 (11)	<.001	120 (36)	269 (16)	<.001	6 (1)	24 (3)	.125
≥60	669 (84)	2347 (89)		209 (64)	1426 (84)		460 (99)	921 (97)	
Level of consciousness, n (%)									
Alert	766 (95)	2629 (100)	<.001	309 (94)	1691 (100)	<.001	457 (96)	938 (99)	<.001
Sleepy/Comatose	38 (5)	13 (0)		20 (6)	5 (0)		18 (4)	8 (1)	
NICU^c^ admission									
Yes	602 (75)	1065 (40)	<.001	262 (80)	819 (48)	<.001	340 (72)	246 (26)	<.001
No	202 (25)	1577 (60)		67 (20)	877 (52)		135 (28)	700 (74)	

Unless otherwise noted, values are N (column %).

a SD, standard deviation.

b PPV, positive pressure ventilation.

c NICU, neonatal intensive care unit.

### Overall Multivariable Model

Overall, among maternal factors (Table 2), any antepartum hemorrhage AHR 1.99 (95% CI: 1.60-2.47) was the predictor of preterm neonatal mortality. Among neonatal factors (Table 3), congenital malformation AHR 3.84 (95% CI: 3.10-4.77), and NICU admission AHR 3.54 (3.02-4.16) were the predictors of preterm neonatal mortality. After combining maternal and neonatal factors (Table 4), resuscitated with PPV, AHR 1.30 (95% CI: 1.08-1.57), congenital malformation, AHR 3.31 (95% CI: 2.64-4.16), and temperature ≤ 35.5°C, AHR 1.18 (95% CI: 1.00-1.39) were predictors of the preterm neonatal mortality.

**Table 2.** Hazard Ratios of Factors Associated With Mortality of Preterm Neonates: Maternal Factors Only.

Characteristic	All		India		Pakistan	
	Unadjusted hazard ratio (95% CI)	Adjusted hazard ratio (95% CI)	Unadjusted hazard ratio (95% CI)	Adjusted hazard ratio (95% CI)	Unadjusted hazard ratio (95% CI)	Adjusted hazard ratio (95% CI)
Maternal factors						
Maternal age (years)						
<20	REF	REF	REF	-	REF	-
20-25	0.78 (0.59, 1.02)	0.87 (0.66, 1.16)	0.94 (0.63, 1.39)	-	0.60 (0.41, 0.86)	-
26-30	1.02 (0.78, 1.35)	0.95 (0.70, 1.29)	1.10 (0.72, 1.68)	-	0.68 (0.47, 0.98)	-
>30	1.18 (0.87, 1.61)	1.03 (0.73, 1.48)	1.15 (0.69, 1.91)	-	0.75 (0.51, 1.11)	-
Maternal education						
No formal schooling	REF	REF	REF	-	REF	REF
<5 years	0.78 (0.54, 1.13)	1.15 (0.78, 1.69)	1.36 (0.75, 2.46)	-	0.79 (0.47, 1.32)	1.03 (0.59, 1.78)
5-12 years	0.57 (0.49, 0.66)	0.82 (0.69, 0.98)	0.89 (0.62, 1.26)	-	0.75 (0.62, 0.91)	1.01 (0.81, 1.24)
>12 years	0.50 (0.36, 0.69)	0.75 (0.53, 1.06)	0.88 (0.54, 1.44)	-	0.59 (0.35, 0.97)	0.79 (0.44, 1.41)
Number of antenatal care visits						
0	REF	REF	REF	-	REF	REF
1-3	0.63 (0.45, 0.89)	1.00 (0.64, 1.59)	0.84 (0.20, 3.60)	-	0.63 (0.44, 0.90)	0.92 (0.57, 1.48)
4+	0.37 (0.27, 0.52)	1.07 (0.68, 1.68)	0.77 (0.19, 3.09)	-	0.53 (0.37, 0.76)	1.17 (0.72, 1.89)
Gravida						
0	REF	REF	REF	-	REF	-
1-3	1.10 (0.94, 1.28)	1.04 (0.88, 1.23)	1.08 (0.86, 1.35)	-	1.00 (0.81, 1.24)	-
4+	1.52 (1.22, 1.89)	0.92 (0.70, 1.21)	1.57 (0.91, 2.72)	-	0.95 (0.74, 1.23)	-
Gestational age (weeks)						
<28.0	REF	REF	REF	REF	REF	REF
28.0-31.6	0.28 (0.23, 0.34)	0.30 (0.24, 0.36)	0.20 (0.15, 0.28)	0.21 (0.16, 0.29)	0.36 (0.28, 0.45)	0.38 (0.29, 0.49)
32.0-36.6	0.04 (0.03, 0.05)	0.05 (0.04, 0.06)	0.03 (0.02, 0.04)	0.03 (0.02, 0.04)	0.06 (0.05, 0.08)	0.06 (0.05, 0.08)
Hemoglobin levels (g/dl)						
<7	REF	REF	REF	-	REF	REF
7-8.9	0.97 (0.64, 1.46)	1.32 (0.87, 2.00)	1.14 (0.55, 2.39)	-	0.73 (0.45, 1.20)	0.98 (0.59, 1.63)
9-10.9	0.77 (0.53, 1.13)	1.01 (0.69, 1.49)	1.18 (0.60, 2.32)	-	0.51 (0.32, 0.82)	0.73 (0.46, 1.18)
11-12.9	0.62 (0.42, 0.91)	0.97 (0.66, 1.44)	0.94 (0.48, 1.85)	-	0.49 (0.31, 0.79)	0.73 (0.45, 1.20)
≥13	0.56 (0.36, 0.86)	0.98 (0.62, 1.54)	1.03 (0.50, 2.10)	-	0.42 (0.22, 0.79)	0.72 (0.38, 1.38)
Any hypertensive disorder	1.04 (0.89, 1.20)	-	1.06 (0.84, 1.32)	-	1.36 (1.10, 1.66)	1.56 (1.25, 1.95)
Any antepartum hemorrhage	1.99 (1.60, 2.47)	1.60 (1.27, 2.02)	1.40 (0.94, 2.07)	-	2.35 (1.81, 3.05)	1.77 (1.33, 2.36)
Mode of delivery						
Vaginal delivery	REF	REF	REF	REF	REF	REF
Cesarean section	0.46 (0.39, 0.55)	0.62 (0.51, 0.75)	0.52 (0.41, 0.67)	0.67 (0.52, 0.86)	0.53 (0.41, 0.69)	0.68 (0.51, 0.90)

**Table 3.** Hazard Ratios of Factors Associated With Mortality of Preterm Neonates: Neonatal Factors Only.

Characteristic	All		India		Pakistan	
	Unadjusted hazard ratio (95% CI)	Adjusted hazard ratio (95% CI)	Unadjusted hazard ratio (95% CI)	Adjusted hazard ratio (95% CI)	Unadjusted hazard ratio (95% CI)	Adjusted hazard ratio (95% CI)
Neonatal factors						
Postnatal age at exam (hours)	1.00 (0.95, 1.05)	-	0.83 (0.71, 0.97)	0.91 (0.81, 1.03)	0.72 (0.54, 0.97)	0.73 (0.54, 0.97)
Sex						
Female	0.84 (0.73, 0.96)	0.85 (0.74, 0.98)	0.80 (0.64, 0.99)	0.84 (0.67, 1.05)	0.85 (0.70, 1.01)	-
Male	REF	REF	REF	REF	REF	-
Birth weight (g)						
<1000	REF	REF	REF	REF	REF	REF
1000-1499	0.30 (0.25, 0.37)	0.59 (0.48, 0.73)	0.24 (0.18, 0.31)	0.55 (0.41, 0.74)	0.46 (0.35, 0.60)	0.70 (0.52, 0.94)
1500-2499	0.06 (0.05, 0.08)	0.21 (0.17, 0.27)	0.04 (0.03, 0.05)	0.16 (0.11, 0.22)	0.09 (0.07, 0.12)	0.27 (0.19, 0.36)
≥2500	0.02 (0.02, 0.03)	0.09 (0.06, 0.13)	0.02 (0.01, 0.04)	0.11 (0.06, 0.21)	0.02 (0.01, 0.03)	0.06 (0.04, 0.11)
Multiple birth	1.42 (1.20, 1.69)	1.18 (0.99, 1.42)	1.36 (1.02, 1.80)	1.14 (0.85, 1.54)	1.37 (1.10, 1.71)	1.10 (0.87, 1.40)
Apgar score (5 minutes)						
0-3	REF	REF	REF	REF	REF	REF
4-6	0.24 (0.20, 0.29)	0.46 (0.37, 0.58)	0.19 (0.14, 0.25)	0.45 (0.32, 0.63)	0.34 (0.27, 0.44)	0.51 (0.39, 0.68)
7-10	0.05 (0.04, 0.05)	0.23 (0.18, 0.29)	0.03 (0.03, 0.05)	0.24 (0.16, 0.37)	0.06 (0.05, 0.08)	0.26 (0.19, 0.36)
Resuscitated with PPV^a^	5.82 (5.03, 6.75)	1.13 (0.95, 1.36)	9.47 (7.61, 11.78)	1.78 (1.34, 2.38)	4.52 (3.65, 5.60)	1.03 (0.80, 1.34)
Congenital malformations	9.33 (7.94, 10.96)	3.84 (3.10, 4.77)	13.86 (10.35, 18.57)	4.27 (2.91, 6.24)	8.78 (7.19, 10.72)	2.70 (2.06, 3.54)
Temperature (°C)						
<35.5	1.29 (1.11, 1.49)	1.04 (0.89, 1.22)	1.48 (1.19, 1.84)	1.09 (0.87, 1.37)	1.65 (1.34, 2.04)	1.28 (1.02, 1.61)
≥35.5	REF	REF	REF	REF	REF	REF
Respiratory rate (breaths per minute)						
<60	0.72 (0.59, 0.87)	0.81 (0.47, 1.13)	0.38 (0.31, 0.48)	0.91 (0.70, 1.19)	1.79 (0.80, 4.01)	-
≥60	REF	REF	REF	REF	REF	-
Level of consciousness						
Alert	0.18 (0.13, 0.25)	0.53 (0.35, 0.81)	0.10 (0.07, 0.16)	0.40 (0.24, 0.68)	0.32 (0.20, 0.52)	0.27 (0.13, 0.57)
Sleepy/comatose	REF	REF	REF	REF	REF	REF
NICU^b^ admission						
Yes	3.54 (3.02, 4.16)	1.10 (0.90, 1.34)	3.60 (2.75, 4.71)	0.88 (0.63, 1.23)	4.57 (3.74, 5.59)	1.55 (1.21, 1.98)
No	REF	REF	REF	REF	REF	REF

a PPV, positive pressure ventilation.

b NICU, neonatal intensive care unit.

**Table 4.** Hazard Ratios of Factors Associated With Mortality of Preterm Neonates: Maternal and Neonatal Factors.

Characteristic	All		India		Pakistan	
	Unadjusted hazard ratio (95% CI)	Adjusted hazard ratio (95% CI)	Unadjusted hazard ratio (95% CI)	Adjusted hazard ratio (95% CI)	Unadjusted hazard ratio (95% CI)	Adjusted hazard ratio (95% CI)
Maternal factors						
Maternal age (years)						
<20	REF	REF	REF	**-**	REF	**-**
20-25	0.78 (0.59, 1.02)	0.89 (0.66, 1.19)	0.94 (0.63, 1.39)	-	0.60 (0.41, 0.86)	-
26-30	1.02 (0.78, 1.35)	0.95 (0.70, 1.31)	1.10 (0.72, 1.68)	-	0.68 (0.47, 0.98)	-
>30	1.18 (0.87, 1.61)	1.12 (0.78, 1.61)	1.15 (0.69, 1.91)	-	0.75 (0.51, 1.11)	-
Maternal education						
No formal schooling	REF	REF	REF	-	REF	
<5 years	0.78 (0.54, 1.13)	1.07 (0.71, 1.62)	1.36 (0.75, 2.46)	-	0.79 (0.47, 1.32)	1.07 (0.59, 1.96)
5-12 years	0.57 (0.49, 0.66)	0.89 (0.73, 1.07)	0.89 (0.62, 1.26)	-	0.75 (0.62, 0.91)	1.17 (0.93, 1.46)
>12 years	0.50 (0.36, 0.69)	0.67 (0.47, 0.97)	0.88 (0.54, 1.44)	-	0.59 (0.35, 0.97)	0.84 (0.45, 1.60)
Number of antenatal care visits						
0	REF	REF	REF	-	REF	
1-3	0.63 (0.45, 0.89)	0.81 (0.51, 1.29)	0.84 (0.20, 3.60)	-	0.63 (0.44, 0.90)	0.78 (0.48, 1.27)
4+	0.37 (0.27, 0.52)	0.69 (0.44, 1.10)	0.77 (0.19, 3.09)		0.53 (0.37, 0.76)	0.79 (0.49, 1.29)
Gravida						
0	REF	REF	REF	-	REF	-
1-3	1.10 (0.94, 1.28)	1.04 (0.88, 1.25)	1.08 (0.86, 1.35)	-	1.00 (0.81, 1.24)	-
4+	1.52 (1.22, 1.89)	0.99 (0.74, 1.33)	1.57 (0.91, 2.72)	-	0.95 (0.74, 1.23)	-
Gestational age (weeks)						
<28.0	REF	REF	REF	REF	REF	REF
28.0-31.6	0.28 (0.23, 0.34)	0.74 (0.58, 0.93)	0.20 (0.15, 0.28)	0.62 (0.44, 0.88)	0.36 (0.28, 0.45)	0.83 (0.61, 1.15)
32.0-36.6	0.04 (0.03, 0.05)	0.36 (0.27, 0.49)	0.03 (0.02, 0.04)	0.40 (0.26, 0.60)	0.06 (0.05, 0.08)	0.46 (0.31, 0.70)
Hemoglobin levels (g/dl)						
<7	REF	REF	REF	-	REF	REF
7-8.9	0.97 (0.64, 1.46)	1.13 (0.72, 1.78)	1.14 (0.55, 2.39)	-	0.73 (0.45, 1.20)	0.77 (0.43, 1.37)
9-10.9	0.77 (0.53, 1.13)	1.06 (0.69, 1.61)	1.18 (0.60, 2.32)	-	0.51 (0.32, 0.82)	0.71 (0.41, 1.23)
11-12.9	0.62 (0.42, 0.91)	0.83 (0.54, 1.28)	0.94 (0.48, 1.85)	-	0.49 (0.31, 0.79)	0.68 (0.39, 1.18)
≥13	0.56 (0.36, 0.86)	0.76 (0.46, 1.23)	1.03 (0.50, 2.10)	-	0.42 (0.22, 0.79)	0.58 (0.28, 1.17)
Any hypertensive disorder	1.04 (0.89, 1.20)	-	1.06 (0.84, 1.32)	-	1.36 (1.10, 1.66)	1.08 (0.85, 1.36)
Any antepartum hemorrhage	1.99 (1.60, 2.47)	1.20 (0.94, 1.53)	1.40 (0.94, 2.07)	-	2.35 (1.81, 3.05)	1.30 (0.95, 1.77)
Mode of delivery						
Vaginal delivery	REF	REF	REF	REF	REF	REF
Cesarean section	0.46 (0.39, 0.55)	0.64 (0.52, 0.78)	0.52 (0.41, 0.67)	0.63 (0.48, 0.82)	0.53 (0.41, 0.69)	0.91 (0.67, 1.25)
Neonatal factors						
Postnatal age at the exam (hours)	1.00 (0.95, 1.05)	-	0.83 (0.71, 0.97)	0.91 (0.82, 1.02)	0.72 (0.54, 0.97)	0.70 (0.50, 0.98)
Sex						
Female	0.84 (0.73, 0.96)	0.86 (0.74, 1.00)	0.80 (0.64, 0.99)	0.85 (0.68, 1.07)	0.85 (0.70, 1.01)	-
Male	REF	REF	REF	REF	REF	-
Birth weight (g)						
<1000	REF	REF	REF	REF	REF	REF
1000-1499	0.30 (0.25, 0.37)	0.76 (0.60, 0.96)	0.24 (0.18, 0.31)	0.79 (0.57, 1.09)	0.46 (0.35, 0.60)	0.77 (0.55, 1.10)
1500-2499	0.06 (0.05, 0.08)	0.42 (0.31, 0.56)	0.04 (0.03, 0.05)	0.29 (0.19, 0.45)	0.09 (0.07, 0.12)	0.44 (0.29, 0.66)
≥2500	0.02 (0.02, 0.03)	0.19 (0.12, 0.31)	0.02 (0.01, 0.04)	0.23 (0.11, 0.48)	0.02 (0.01, 0.03)	0.12 (0.06, 0.22)
Multiple birth	1.42 (1.20, 1.69)	1.09 (0.90, 1.34)	1.36 (1.02, 1.80)	1.00 (0.75, 1.35)	1.37 (1.10, 1.71)	1.15 (0.88, 1.51)
Apgar score (5 minutes)						
0-3	REF	REF	REF	REF	REF	REF
4-6	0.24 (0.20, 0.29)	0.50 (0.40, 0.63)	0.19 (0.14, 0.25)	0.44 (0.31, 0.63)	0.34 (0.27, 0.44)	0.55 (0.40, 0.74)
7-10	0.05 (0.04, 0.05)	0.27 (0.21, 0.36)	0.03 (0.03, 0.05)	0.25 (0.16, 0.38)	0.06 (0.05, 0.08)	0.29 (0.20, 0.42)
Resuscitated with PPV^a^	5.82 (5.03, 6.75)	1.30 (1.08, 1.57)	9.47 (7.61, 11.78)	1.76 (1.32, 2.35)	4.52 (3.65, 5.60)	1.05 (0.78, 1.39)
Congenital malformations	9.33 (7.94, 10.96)	3.31 (2.64, 4.16)	13.86 (10.35, 18.57)	3.92 (2.67, 5.76)	8.78 (7.19, 10.72)	2.58 (1.93, 3.45)
Temperature (°C)						
<35.5	1.29 (1.11, 1.49)	1.18 (1.00, 1.39)	1.48 (1.19, 1.84)	1.15 (0.91, 1.45)	1.65 (1.34, 2.04)	1.32 (1.04, 1.69)
≥35.5	REF	REF	REF	REF	REF	REF
Respiratory rate (breaths per minute)						
<60	0.72 (0.59, 0.87)	0.40 (0.12, 0.74)	0.38 (0.31, 0.48)	0.90 (0.69, 1.18)	1.79 (0.80, 4.01)	-
≥60	REF	REF	REF	REF	REF	-
Level of consciousness						
Alert	0.18 (0.13, 0.25)	0.42 (0.27, 0.66)	0.10 (0.07, 0.16)	0.41 (0.24, 0.71)	0.32 (0.20, 0.52)	0.25 (0.12, 0.53)
Sleepy/comatose	REF	REF	REF	REF	REF	REF
NICU^b^ admission						
Yes	3.54 (3.02, 4.16)	1.06 (0.86, 1.31)	3.60 (2.75, 4.71)	1.03 (0.73, 1.45)	4.57 (3.74, 5.59)	1.39 (1.06, 1.82)
No	REF	REF	REF	REF	REF	REF

a PPV, positive pressure ventilation.

b NICU, neonatal intensive care unit.

### Multivariable Model for India

After combining maternal and neonatal factors (Table 4), resuscitated with PPV, AHR 1.76 (95% CI: 1.32-2.35), and congenital malformation, AHR 3.92 (95% CI: 2.67-5.76) were predictors of the preterm neonatal mortality.

### Multivariable Model for Pakistan

After combining maternal and neonatal factors (Table 4), congenital malformation, AHR 2.58 (95% CI: 1.93-3.45), temperature ≤35.5°C, AHR 1.32 (95% CI: 1.04-1.69), and NICU admission AHR, 1.39 (95% CI: 1.06-1.82) were predictors of the preterm neonatal mortality.

## Discussion

In this study, we found that the frequency of preterm neonatal death is twice as high in Pakistan compared to India. Overall, preterm neonates born to mothers with any antepartum hemorrhage were more likely to die whereas among neonatal factors, congenital malformation, temperature <35.5°C and NICU admission were the predictors of preterm neonatal mortality. In Pakistan, preterm neonates born to mothers with any hypertensive disorder and any antepartum hemorrhage were more likely to die. Furthermore, when both maternal and neonatal factors were combined in a model, none of the maternal factors predicted preterm neonatal death in India and Pakistan separately and combined.

The findings of this study are consistent with previous studies that showed that pregnancy outcomes are poor in Pakistan as compared to other LMICs. However, it is difficult to identify which maternal and neonatal factors are responsible for the high mortality in Pakistan.^21^ There appears to be a complex interplay of several maternal, neonatal, and healthcare-related factors that contribute to these poor outcomes. Although some of the factors that have been identified in previous studies are attributed to the low level of literacy, short inter-delivery intervals, and high parity among Pakistani women which are consistent with our study as well.^21^

Furthermore, in this study, the maternal factors predicting preterm neonatal deaths was found to be less important, which is similar to studies done in other developing countries. A study conducted in Ghana reported that neonatal and household factors have more predictive accuracy for preterm neonatal deaths within 28 days as compared to maternal factors.^22^ Other studies suggest that neonatal factors are more predictive of neonatal death as compared to maternal factors alone. We also found that overall, preterm neonates with congenital malformations were more likely to die. In India, preterm neonates who were resuscitated with PPV and congenital malformations were more likely to die, whereas in Pakistan preterm neonates with congenital structural malformation, temperature <35.5°C, and preterm neonates admitted to NICU were more likely to die. Congenital malformations are one of the major contributing factors to prematurity and neonatal deaths globally. According to the WHO mortality database, 17% to 42% of infant mortality is attributed to congenital malformation, and 9 out of 10 neonates with severe congenital malformations come from LMICs. Although in this paper we haven’t stratified the type of congenital malformations, the presence of a congenital malformation is an important predictor of neonatal death both in Pakistan and India.

Additionally, temperature <35.5°C was a predictor of neonatal mortality. This result is similar to another study that reported that neonates who had temperature <35.5°C at admission were 2.6 times more at risk of death as compared to normothermic neonates. Also, temperature <35.5°C could be a symptom of underlying sepsis or infection which is also a predictor of neonatal mortality.^23,24^ A similar study conducted in Ethiopia found that neonates admitted to the NICU or temperature <35.5°C were more likely to die.^25^

Moreover, in low-resource settings like Pakistan and India, not only neonatal factors but also the quality and availability of healthcare services in hospitals have a complex direct or indirect relationship with these predictors. Scarcity of resources and a lack of trained staff are among the contributory factors for these deaths.^26-28^ Our study also revealed that survival rates decline more rapidly in Pakistan than in India. These findings in Pakistan can be attributed to issues of healthcare access and quality, as well as a lack of a national policy on neonatal health.^29^

Preterm neonates resuscitated with positive pressure ventilation, congenital malformations, or temperature <35.5°C, were more likely to die, irrespective of country (India vs Pakistan). The above findings would be helpful to provide insight into the important predictors for preterm neonatal mortality, especially in India and Pakistan. Because of the above findings, we recommend that facilities in low-resource settings should be equipped to identify these predictors and should be able to provide appropriate resuscitation care.^30^ These predictors can also be utilized for devising context-specific interventions and flagging infants for continuous monitoring. We further suggest that access to high-quality intrapartum and postnatal care is of utmost importance to improve neonatal outcomes. Also, community workers can be trained further to identify these predictors and to closely follow up on these preterm infants.^3,30,31^

### Strengths

First, the data were used from a prospective observational study from India and Pakistan on predictors of preterm neonatal deaths. Second, the GA was calculated using a gestational age calculator with an algorithm adjusted for the last menstrual period and/or antenatal ultrasound and/or Ballard scoring. Third, an advanced statistical analysis modeling with separate models as well as the combined model for maternal factor and neonatal factors.

### Limitations

This study has some limitations. Firstly, the study’s generalizability is limited because this study was conducted in tertiary care referral hospitals only. Secondly, this study did not have data on maternal nutritional status, and community and environmental factors as well as variables such as skin-to-skin contact, antenatal corticosteroid use, antenatal fetal monitoring, and temperature at the time of admission that might help to predict neonatal mortality. Third, since we utilized data from a large observational study, we did not calculated the sample size. Lastly, since we calculated post hoc power, it’s important to note that it relies on observed data, potentially leading to biased results as it may not accurately reflect the underlying assumptions made during the study design.

This study has clinical and public health implications. The model aids early identification of high-risk infants, informing targeted interventions based on maternal factors like antepartum hemorrhage and neonatal factors such as positive pressure ventilation. The findings guide resource allocation in healthcare and inform policy development, emphasizing the importance of tailored antenatal care and health education programs.

## Conclusion

In conclusion, this study highlights key determinants of preterm neonatal mortality in both maternal and neonatal contexts, emphasizing the need for targeted interventions. Beyond clinical applications, collaborative public health efforts are crucial to addressing disparities and navigating regional variations. Informed decision-making, resource allocation, and ongoing research are pivotal in mitigating the challenges of preterm neonatal mortality.

## Supplemental Material

sj-docx-1-gph-10.1177_2333794X241236617 – Supplemental material for Predictors of Preterm Neonatal Mortality in India and Pakistan: A Secondary Analysis of Data from PURPOSe Study Supplemental material, sj-docx-1-gph-10.1177_2333794X241236617 for Predictors of Preterm Neonatal Mortality in India and Pakistan: A Secondary Analysis of Data from PURPOSe Study by Shiyam Sunder Tikmani, Sarah Saleem, Afreen Sadia, Carla M. Bann, Muhammad Hayat Bozdar, Jamal Raza, Sangappa M. Dhaded, Shivaprasad S. Goudar, Guruparasad Gowdar, Haleema Yasmin, Elizabeth M. McClure and Robert L. Goldenberg in Global Pediatric Health
