# Supplementary material for: Predictors of Preterm Neonatal Mortality in India and Pakistan: A Secondary Analysis of Data from PURPOSe Study
Source: Glob Pediatr Health. 2024 Mar 13;11:2333794X241236617. doi: 10.1177/2333794X241236617 (PMC10938607; doi:10.1177/2333794X241236617)
Supplement: sj-docx-1-gph-10.1177_2333794X241236617 – Supplemental material for Predictors of Preterm Neonatal Mortality in India and Pakistan: A Secondary Analysis of Data from PURPOSe Study [file sj-docx-1-gph-10.1177_2333794X241236617.docx]

**Supplementary Table 1: Post hoc power calculation for predictors of preterm neonatal mortality**

| **Characteristics** | | **All** | | | | | | **India** | | | | | **Pakistan** | | | |
| --- | --- | --- | --- | --- | --- | --- | --- | --- | --- | --- | --- | --- | --- | --- | --- | --- |
| 1. **Maternal factor** | | | | | | | | | | | | | | | | |
|  | | HR | | N | | Power | | HR | | N | | Power | HR | | N | Power |
| Any antepartum hemorrhage | | 1.60 | | 3446 | | 1.0 | | - | | - | | - | 1.77 | | 1421 | 1.0 |
| 1. **Neonatal factors** | | | | | | | | | | | | | | | | |
| Resuscitated with PPV | | - | | - | | - | | 1.78 | | 2025 | | 1.0 | - | | - | - |
| Congenital structural abnormalities | | 3.84 | | 3446 | | 1.0 | | 4.27 | | 2025 | | 1.0 | 2.70 | | 1421 | 1.0 |
| Temperature (^o^C)  <35.5  ≥ 35.5 | | - | | - | | - | | - | | - | | - | 1.28  Ref | | 1421 | 0.94 |
| Respiratory rate (breaths per minute)  <60  ≥60 | | 1.81  Ref | | 3446 | | 1.0 | | - | | - | | - | - | | - | - |
| NICU admission  Yes  No | | - | | - | | - | | - | | - | | - | 1.55  Ref | | 1421 | 1.0 |
| 1. **Maternal and neonatal factors (Combined)** | | | | | | | | | | | | | | | | |
| Resuscitated with PPV | 1.30 | | 3446 | | 1.0 | | 1.76 | | 2025 | | 1.0 | | - | - | | - |
| Congenital structural abnormalities | 3.31 | | 3446 | | 1.0 | | 3.92 | | 2025 | | 1.0 | | 2.58 | 1421 | | 1.0 |
| Temperature (^o^C)  <35.5  ≥ 35.5 | 1.18  Ref | | 3446 | | 0.9 | | - | | - | | - | | 1.32  Ref | 1421 | | 0.97 |
| Respiratory rate (breaths per minute)  <60  ≥60 | 1.40  Ref | | 3446 | | 1.0 | | - | | - | | - | | - | - | | - |
| NICU admission  Yes  No | - | | - | | - | | - | | - | | - | | 1.39  Ref | 1421 | | 1.0 |
